# Preparation and Modification of Silicalite-2 Membranes

**DOI:** 10.3390/membranes15020054

**Published:** 2025-02-08

**Authors:** Yin Yang, Juan Liu, Qi Zhou, Siqi Shao, Lingling Zou, Wenjun Yuan, Meihua Zhu, Xiangshu Chen, Hidetoshi Kita

**Affiliations:** 1 State-Province Joint Engineering Laboratory of Zeolite Membrane Materials, School of Chemical Engineering, Jiangxi Normal University, Nanchang 330022, China; yangyin@jxnu.edu.cn (Y.Y.); liujuan@jxnu.edu.cn (J.L.); zhouqi@jxnu.edu.cn (Q.Z.); shaosiqi@jxnu.edu.cn (S.S.); zoulingling@jxnu.edu.cn (L.Z.); yuanwenjun@jxnu.edu.cn (W.Y.); 2 Graduate School of Science and Technology for Innovation, Graduate School Science and Engineering, Yamaguchi University, Ube 755-8611, Japan

**Keywords:** Silicalite-2 membrane, pervaporation, trimethylchlorosilane, modification

## Abstract

Silicalite-2 membranes were successfully prepared on tubular α-Al_2_O_3_ supports by secondary hydrothermal synthesis, and the pervaporation performance of the membrane was evaluated by separation of a 5 wt% ethanol/H_2_O mixture at 60 °C. The effects of templating agent content, water–silicon ratio and crystallization time on the separation performance of Silicalite-2 membranes were investigated. When the TBAOH/SiO_2_ and H_2_O/SiO_2_ molar ratios of the precursor synthesis solution were 0.2 and 120, a dense Silicalite-2 membrane could be prepared on the surface of the tubular α-Al_2_O_3_ support after 72 h. The silane coupling agent was utilized to treat the Silicalite-2 membranes, and the effects of silane coupling agent dosage on their properties were also explored. The pervaporation performance of the Silicalite-2 membrane was greatly improved with a 5.7 wt% trimethylchlorosilane (TMCS) solution and the flux and separation factor of the membrane reached 1.75 kg·m^−2^·h^−1^ and 22 for separation of 5 wt% EtOH/H_2_O at 60 °C, respectively.

## 1. Introduction

Fuel ethanol, as a renewable energy source, has garnered significant attention due to the escalating concerns about environmental protection and the energy crisis [1,2,3]. At present, fermentation is key for producing fuel ethanol [4]. Improving efficiency and reducing the energy consumption of fermentation are important tasks in the production of ethanol. Kaseno et al. [5] found that continuously removing ethanol retained the high productivity of the fermentation process. Pervaporation (PV) is considered as one of the most promising energy-efficient processes [6,7]. In PV application, excellent membrane materials are important. Zeolite membranes have unique advantages, such as high temperature resistance, corrosion resistance, regular pore structure and excellent adsorption selection, which are promising alternative membranes for PV application [8,9,10]. Wu et al. [11] utilized hydrophobic MFI zeolite membranes to enrich butanol from an acetone–butanol–ethanol (ABE) solution, which is a promising process for producing biobutanol.

Silicalite membranes (Silicalite-1 and Silicalite-2), which are free of aluminium and have high hydrophobicity, play a significant role in the condensation of a low-concentration ethanol solution [12]. Lan et al. [13] prepared Silicalite-1 membranes for separating a 3 wt% ethanol/H_2_O solution mixture. The separation factor and flux achieved were as high as 79 and 1.40 kg·m^−2^·h^−1^, respectively. Li et al. [14] prepared silanol group-free Silicalite-1 membranes that showed a stable separation performance over 80 h.

Silicalite-2 is an MEL-type zeolite and has straight a-axis and b-axis channels, which are favorable for molecular diffusion and application of liquid separation [15]. Kosinov et al. [16] prepared thin and pure Silicalite-2 membranes on porous α-alumina hollow fiber supports. The separation factor and the flux were 17.2 and 3.60 kg·m^−2^·h^−1^ for the 5 wt% ethanol/H_2_O mixture at 60 °C. Gong et al. [17] prepared Silicalite-2 membranes using two seed crystals with different sizes. The optimal separation factor and flux were 6.7 and 7.61 kg·m^−2^·h^−1^ for the 5 wt% ethanol/H_2_O mixture at 60 °C. Wu et al. [18] prepared Silicalite-2 membranes on mesoporous α-Al_2_O_3_ supports by secondary hydrothermal synthesis; the separation factor and flux were 5.1 and 1.12 kg·m^−2^·h^−1^ for a 5 wt% ethanol/H_2_O mixture at 70 °C. In addition, Table 1 summarized the pervaporation (PV) performance of Silicalite-2 membranes for a 5 wt% ethanol/H_2_O mixture, where the separation performance was poor.

However, the preparation of dense Silicalite-2 membranes is difficult and modification is an effective method to change the hydrophobic features and improve the separation performance of the zeolite membrane. Ren et al. modified Silicalite-1 membranes with silane coupling agents and improved the separation performance [21]. Fariba et al. [22] prepared and modified Silicalite-1 membranes with polydimethylsiloxane and a dual active layer (superhydrophobic (TALS) Silicalite-1/(PDMS) membranes) was formed by the spraying method. The flux and separation factor of the membrane were 1.88 kg·m^−2^·h^−1^ and 32.34 for the 5 wt% ethanol/H_2_O solution. Kosinov et al. [19] modified the surface of Silicalite-2 membranes with triethoxysilafluorosilane to improve the separation factor of the membrane.

Herein, Silicalite-2 membranes were successfully prepared on α-Al_2_O_3_ supports by a secondary hydrothermal process. The effects of the TBAOH (tetrabutylammonium hydroxide)/SiO_2_ ratio, H_2_O/SiO_2_ ratio of the precursor synthesis solution and crystallization time on the growth of Silicalite-2 membranes were investigated. In addition, PV performance of the membrane was modified and improved with a silane coupling agent (TMCS). The optimal TMCS concentration was also investigated in this work.

## 2. Experimental Section

### 2.1. Experimental Materials

Silicalite-2 membranes were prepared on α-Al_2_O_3_ tubular supports (OD. 12 mm, ID. 9 mm, length. 10 cm, Kailai Chemical, Yixing, China). The silica source and organic templates included tetraethyl orthosilicate (TEOS, 99%, Innochem, Pyeongtaek-si, Republic of Korea) and TBAOH (25%, Alaadin, Fukuoka, Japan). Ammonia (25%, Aladdin) and trimethylchlorosilane (TMCS, 98%, Aladdin) were used to improve the hydrophobicity of Silicalite-2 membranes.

### 2.2. Preparation of Silicalite-2 Zeolites

Silicalite-2 zeolites were prepared by in situ hydrothermal processes. The molar composition of the precursor synthesis solution was 1TEOS: 0.25TBAOH: 20H_2_O. TBAOH and TEOS were mixed with water and hydrolyzed at room temperature for 24 h. Then, the mixture was transferred to a stainless-steel autoclave at 130 °C for 48 h. Finally, Silicalite-2 zeolites were obtained by washing, centrifugation, drying and calcination at 550 °C for 10 h.

### 2.3. Preparation of Silicalite-2 Membranes

Silicalite-2 membranes were prepared by secondary hydrothermal synthesis. The molar composition of the precursor solutions was 1TEOS: x TBAOH: yH_2_O (x = 0.15~0.30, y = 120~240). The details of the synthesis conditions of Silicalite-2 membranes are summarized in Table 2. TBAOH, TEOS and de-ionized water were mixed and stirred continuously at room temperature to form a clarified solution. The Silicalite-2 seed crystals were attached to the outer surface of α-Al_2_O_3_ tubular supports by a rubbing coating. The seeded support and transparent synthesis solution were loaded into a stainless-steel autoclave, and crystallization continued at 150 °C for 48, 72, and 96 h, respectively. The membranes were washed with water after crystallization. Thereafter, calcination of the Silicalite-2 membranes was carried out at 500 °C to remove the organic templating agent.

### 2.4. Modification of Silicalite-2 Membrane

Modification of Silicalite-2 membrane was carried out as follows: TEOS, ammonia, ethanol and water were mixed and formed a silica solution at 50 °C for 2 h. Silicalite-2 membranes were sealed at both ends and submerged into the silica solution for 30 s, and then the membranes were dried and calcined at 450 °C for 1 h. Then, the Silicalite-2 membranes were placed into the mixture of TMCS and ethanol at 50 °C for 5 h and the TMCS concentration in the mixture ranged from 0 to 7.5 wt%.

### 2.5. Characterization and PV Performance of Silicalite-2 Membranes

X-ray diffraction (XRD) patterns of the samples were recorded on a Rigaku Ultima IV diffractometer using Cu Kα radiation (λ = 0.15405 nm, 40 kV, and 40 mA) at a scanning rate of 6°·minQ^−1^ and the 2θ angle range was 5~50°. Morphology and crystal size of the Silicalite-2 membranes were determined by a field emission scanning electron microscope (FE-SEM, Hitachi SU8020, Tokyo, Japan). Hydrophobicity/hydrophilicity of the membrane was analyzed using contact angle equipment (HKCA-10, Haker, Nanjing, China).

**Figure 1:**
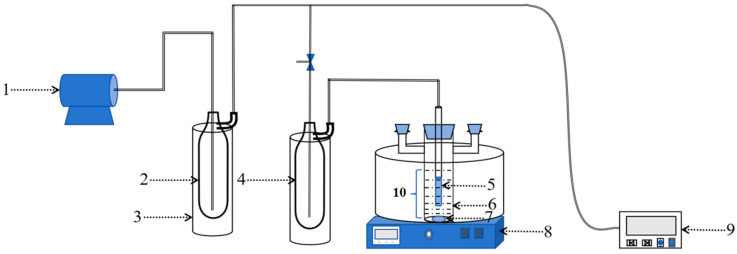
Schematic diagram of PV device. 1: Vacuum pump; 2: safety bottle; 3: liquid nitrogen bottle; 4: collecting bottle; 5: Silicalite-2 membrane (OD. 12 mm, ID. 9 mm, Length. 10 cm); 6: reactor; 7: magnet; 8: bath oil pots; 9: vacuum gauge; 10: feed mixture.

The PV performance of Silicalite-2 membranes was tested using 5 wt% ethanol/H_2_O solution at 60 °C. A schematic diagram of the PV device is presented in Figure 1. Permeation was collected at the fixed time intervals, and gas chromatography (GC-2014C, Shimadzu, Kyoto, Japan) was applied to analyze the composition of the permeation. Pervaporation performance was evaluated by two parameters: permeate flux Q (kg·m^−2^·h^−1^) and separation factor (α). The flux and the separation factor were calculated by the following formula:

(1) $$Q=\frac{W}{t\times A}$$

(2) $$\alpha =\frac{y_{a}/y_{b}}{x_{a}/x_{b}}$$

where W is the total mass of the permeate side (kg), A is the effective membrane area (m^2^), and t is the time interval (h). *y_a_*, *y_b_*, *x_a_* and *x_b_* are the contents of the permeation and feed mixture, respectively.

## 3. Results and Discussion

### 3.1. Effect of TBAOH/SiO_2_ Molar Ratio

**Figure 2:**
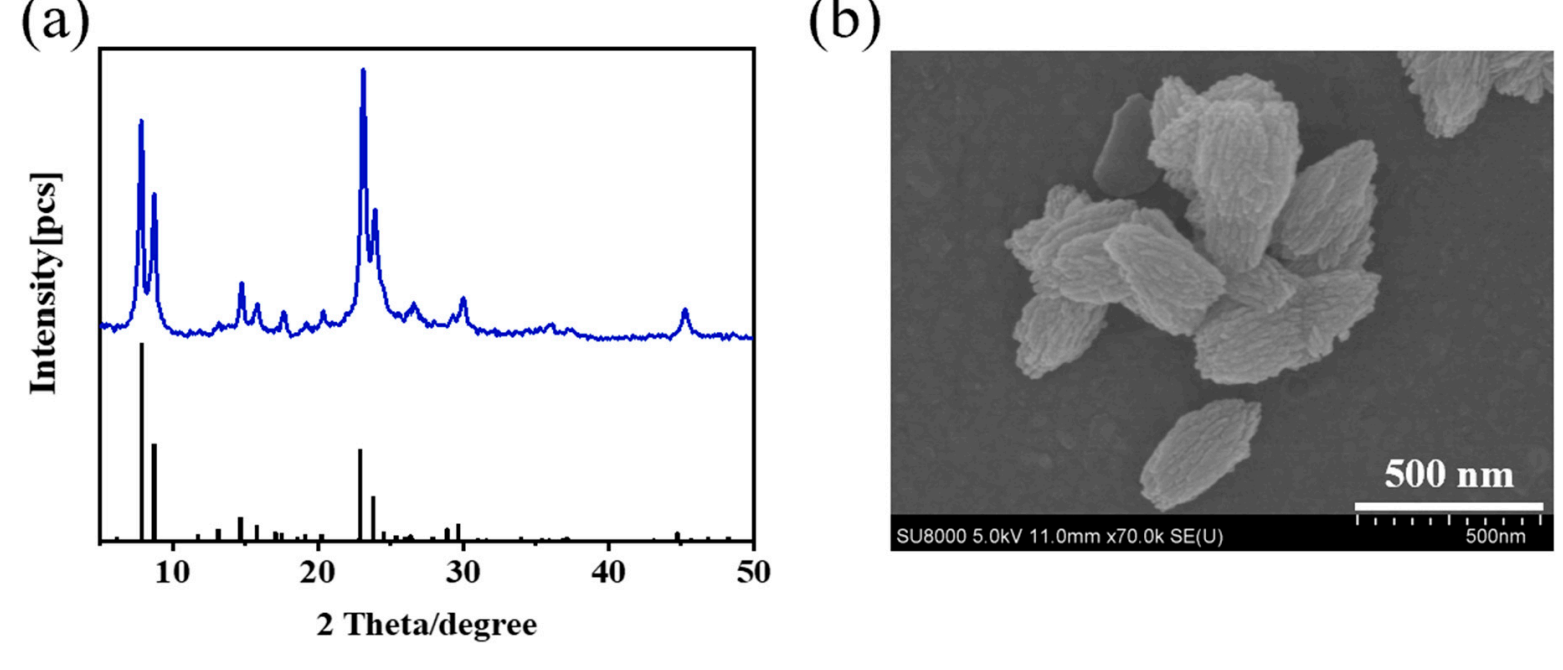
XRD patterns (**a**) and SEM image (**b**) of Silicalite-2 zeolites.

XRD patterns and FE-SEM images of Silicalite-2 zeolites are shown in Figure 2. The XRD pattern exhibited five typical diffraction peaks for MEL zeolites at 2θ values of 7.9°, 8.8°, 23.1°, 23.9°, and 45.2°. SEM images revealed that the Silicalite-2 zeolites had a walnut-like shape, and the zeolite crystal size was around 400–500 nm.

**Figure 3:**
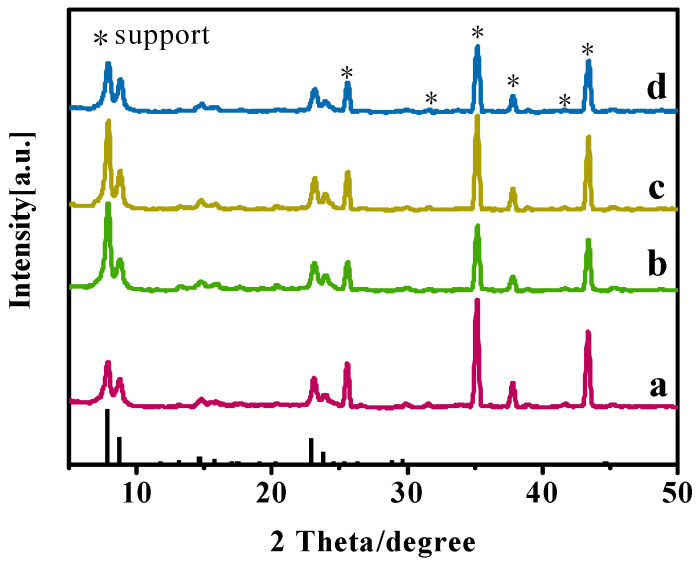
XRD patterns of Silicalite-2 membranes with different TBAOH/SiO_2_ ratios: C-1 TBAOH/SiO_2_ = 0.15 (a), C-2 TBAOH/SiO_2_ = 0.20 (b), C-3 TBAOH/SiO_2_ = 0.25 (c) and C-4 TBAOH/SiO_2_ = 0.30 (d).

**Figure 4:**
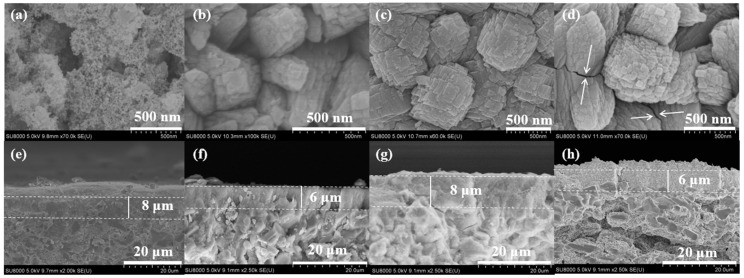
Surface and cross sectional SEM images of Silicalite-2 membranes with different TBAOH/SiO_2_ ratios: C-1 TBAOH/SiO_2_= 0.15 (**a**,**e**), C-2 TBAOH/SiO_2_ = 0.2 (**b**,**f**), C-3 TBAOH/SiO_2_ = 0.25 (**c**,**g**) and C-4 TBAOH/SiO_2_ = 0.3 (**d**,**h**).

Organic templating agents played a crucial role in the nucleation and crystallization of silicalites or silicalite membranes [23]. In this study, the effects of the TBAOH/SiO_2_ molar ratio on the growth of Silicalite-2 membranes (C-1, C-2, C-3, and C-4) were investigated. As summarized in Table 2, the TBAOH/SiO2 molar ratios of the precursor synthesis solution were 0.15, 0.20, 0.25, and 0.30. XRD patterns and SEM images of the membranes are shown in Figure 3 and Figure 4. Although the TBAOH/SiO_2_ molar ratios varied from 0.15 to 0.30, all membranes exhibited five characteristic diffraction peaks of MEL zeolites (2θ = 7.9°, 8.8°, 23.1°, 23.9°, and 45.2°), confirming that the Silicalite-2 membranes were successfully prepared in this study [24].

Additionally, surface SEM images demonstrate that the TBAOH/SiO_2_ molar ratio significantly influenced the growth of the Silicalite-2 membranes. When the TBAOH/SiO_2_ molar ratio of the precursor synthesis solution was 0.15, few fine crystals were observed on the support surface, and a large amount of amorphous covered the support (Figure 4a,e), the amount of amorphous material decreased with high TBAOH/SiO_2_ molar ratio, the fine Silicalite-2 crystals grew into a walnut-like shape, and formed a continuous zeolite layer on the support surface (Figure 4b–d,f–h). However, when the TBAOH content in the initial synthesis gel was excessive, cracks appeared on the surface of the Silicalite-2 membrane (C-4) (Figure 4d).

### 3.2. Effect of H_2_O/SiO_2_ Molar Ratio

**Figure 5:**
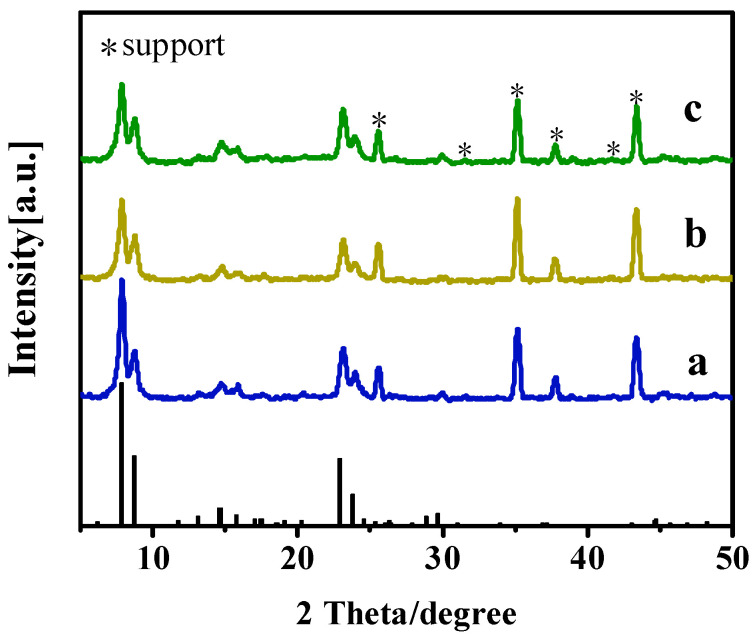
XRD patterns of Silicalite-2 membranes with different H_2_O/SiO_2_ molar ratios: C-5, H_2_O/SiO_2_ = 120 (a), C-6, H_2_O/SiO_2_ = 200 (b) and C-7, H_2_O/SiO_2_ = 240 (c).

**Figure 6:**
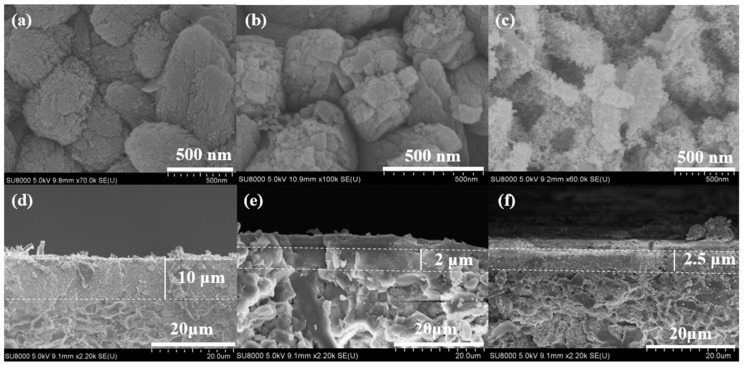
Surface and cross sectional SEM images of Silicalite-2 membranes with different H_2_O/SiO_2_ ratios: C-5, H_2_O/SiO_2_ = 120 (**a**,**d**), C-6, H_2_O/SiO_2_ = 200 (**b**,**e**) and C-7, H_2_O/SiO_2_ = 240 (**c**,**f**).

The water content of the precursor synthesis solution played a crucial role in heat transfer and significantly influenced the growth of zeolite membranes [25]. In this study, Silicalite-2 membranes (C-5, C-6, and C-7) were prepared using different H_2_O/SiO_2_ molar ratios. H_2_O/SiO_2_ molar ratios of the precursor synthesis solution were 120, 200, and 240. XRD patterns and SEM images of the membranes are presented in Figure 5 and Figure 6.

All Silicalite-2 membranes exhibited the characteristic peaks of the MEL zeolite structure. However, crystallinity of the membranes gradually decreased with increasing water content in the precursor synthesis solution, which was consistent with the SEM images. When the H_2_O/SiO_2_ molar ratio of the initial synthesis solution was 120, closely arranged walnut-like crystals formed, resulting in a dense zeolite layer on the support surface (Figure 6a,d). As the H_2_O/SiO_2_ molar ratio increased to 240, a greater number of fine crystals and amorphous material appeared on the support surface, and the thickness of the zeolite layers decreased (Figure 6b,c,e,f). Hence, the highH_2_O/SiO_2_ ratios resulted in a thin zeolite layer and less defined crystal structures. Precise control of the water content was essential for optimizing the membrane’s structural integrity and performance.

### 3.3. Effect of Crystallization Time

**Figure 7:**
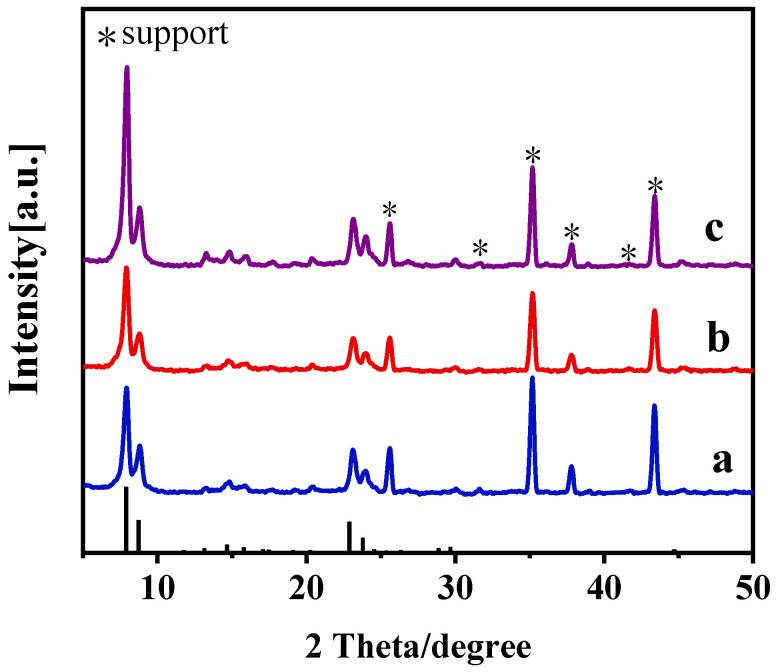
XRD patterns of Silicalite-2 membranes with different crystallization times: (a) C-8, 48 h, (b) C-9, 72 h and (c) C-10, 96 h.

**Figure 8:**
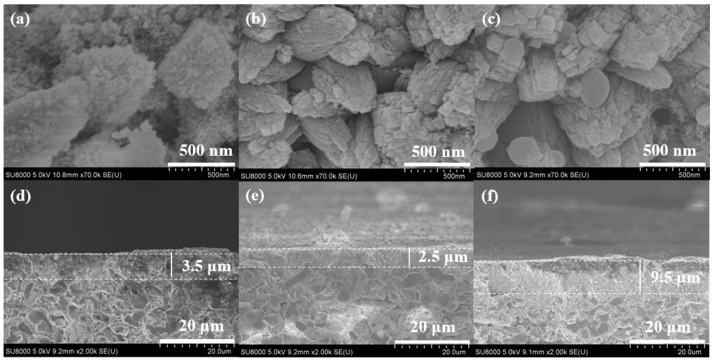
Surface and cross sectional SEM images of Silicalite-2 membranes with different crystallization times: C-8, 48 h (**a**,**d**); C-9, 72 h (**b**,**e**); and C-10 6 h (**c**,**f**).

Crystallization time was crucial for preparing a compact zeolite membrane and significantly affected the crystallinity of the zeolite membranes [26]. In this study, the effects of crystallization time (C-8, C-9, and C-10) on the growth of Silicalite-2 membranes were explored, with crystallization times of 48, 72, and 96 h. XRD patterns and SEM images of the membranes are presented in Figure 7 and Figure 8.

All C-8, C-9, and C-10 membranes exhibited the typical diffraction characteristic peaks of MEL zeolites, indicating successful synthesis. The crystallinity of the zeolite membranes increased with long crystallization times. When the synthesis time was 48 h, only fine crystals were observed on the support (Figure 8a,d). When the crystallization time was extended to 72 h, inter-grown walnut-like Silicalite-2 zeolites formed a dense zeolite layer on the support surface (Figure 8b,e). There large zeolite crystals formed a thick zeolite layer on the surface after 96 h (Figure 8c,f). A thicker zeolite layer may affect the permeability and performance of the membrane. Therefore, the crystallization time should be carefully controlled to achieve the optimal PV performance.

### 3.4. Influences of Trimethylchlorosilane Content

**Figure 9:**
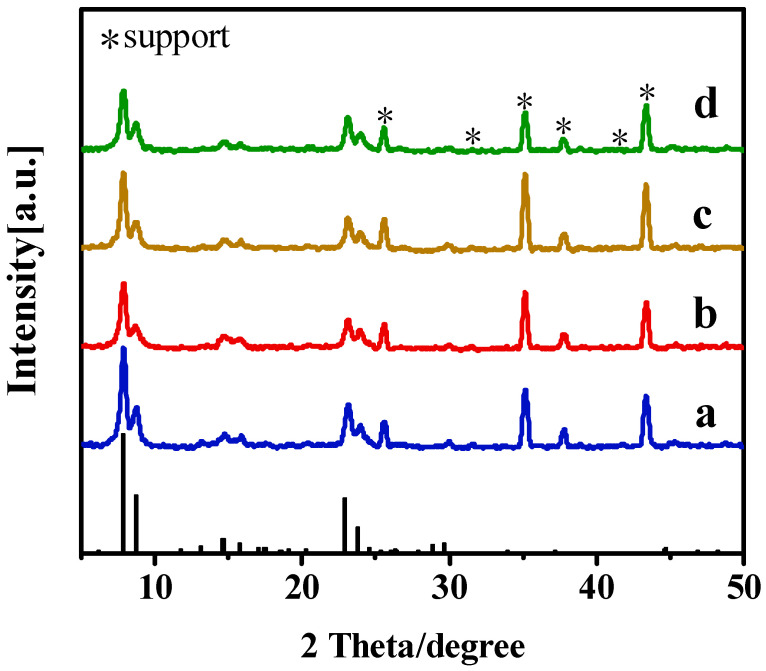
XRD patterns of the modified Silicalite-2 membranes with different TMCS contents: C-11, 0 wt% TMCS (a); C-12, 3.9 wt% TMCS (b); C-13, 5.7 wt% TMCS (c); C-14, 7.5 wt% TMCS (d).

**Figure 10:**
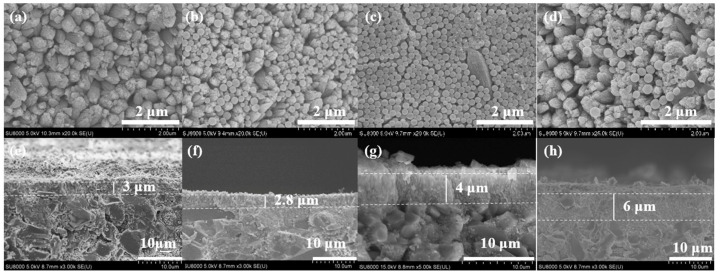
Surface and cross sectional SEM images of Silicalite-2 membranes and modified Silicalite-2 membranes with different TMCS contents: C-11, 0 wt% TMCS (**a**,**e**); C-12, 3.9 wt% TMCS (**b**,**f**); C-13, 5.7 wt% TMCS (**c**,**g**); C-14, 7.5 wt% TMCS (**d**,**h**).

**Figure 11:**
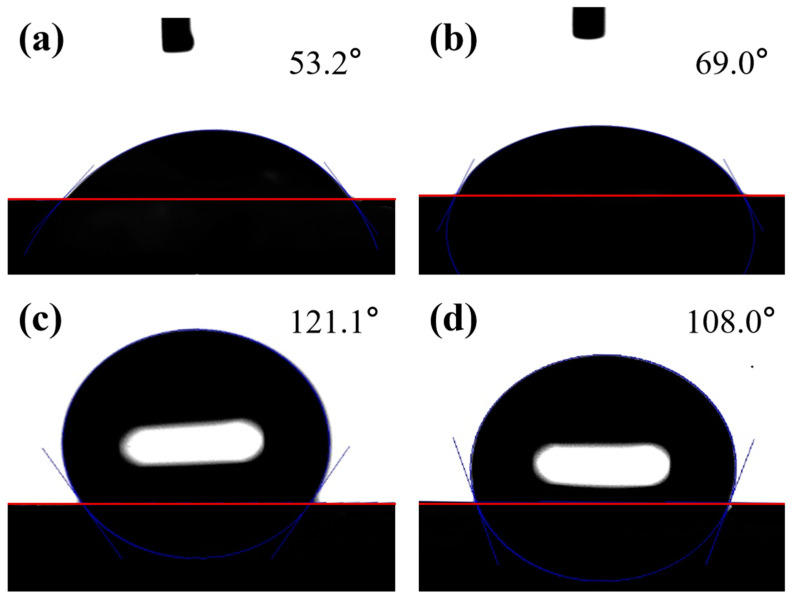
Water contact angles of Silicalite-2 membrane and modified Silicalite-2 membrane with different TMCS contents: C-11, 0 wt% TMCS (**a**); C-12, 3.9 wt% TMCS (**b**); C-13, 5.7 wt% TMCS (**c**) and C-14, 7.5 wt% TMCS (**d**).

Generally, the hydrophobicity of the zeolite membranes can be modified and improved by a silane coupling agent, which attaches to the zeolite’s surface during a silicon hydroxyl reaction [27]. As shown in Table 3, the Silicalite-2 membranes (C-11) had poor PV performance for separation of the 5 wt% EtOH/H_2_O mixture at 60 °C and the separation factor of the membrane was only 3, which was attributed to the presence of numerous inter-crystalline gaps within the membrane layer. The effects of TMCS content (3.9%, 5.7% and 7.5%) on the PV performance and morphology of the membranes (C-12, C-13 and C-14) were investigated in this work. As shown in Figure 9, XRD patterns of the modified membranes showed the typical diffraction peaks of MEL zeolites. SEM images of the modified membranes presented demonstrated that various spherical pellets were formed and covered the surface of Silicalite-2 membranes, and the amount of spherical pellets increased with TMCS content (Figure 10). In addition, Figure 11 shows the water contact angles of modified Silicalite-2 membranes with different TMCS contents. The water contact angle of the modified zeolite membrane was significantly larger than that of the original Silicalite-2 membrane, and the contact angle increased with increasing TMCS content, indicating that TMCS modification could improve the hydrophobicity of the Silicalite-2 membranes in this work. The excessive TMCS molecules may cause steric hindrance and lead to a less ordered arrangement of -OSi(CH3)3 groups on the membrane surface, as shown in Table 3. When the TMCS content increased to 7.5%, the water contact angle decreased from 121.1° to 108° and the surface became less uniformly covered with hydrophobic groups, reducing the overall hydrophobicity of the membrane. The PV performance of the modified membranes, especially the separation factor, was greatly improved by TMCS modification. For example, the separation factor and total flux of the membrane C-13 were 22 and 1.75 kg·m^−2^·h^−1^, respectively [28].

**Figure 12:**
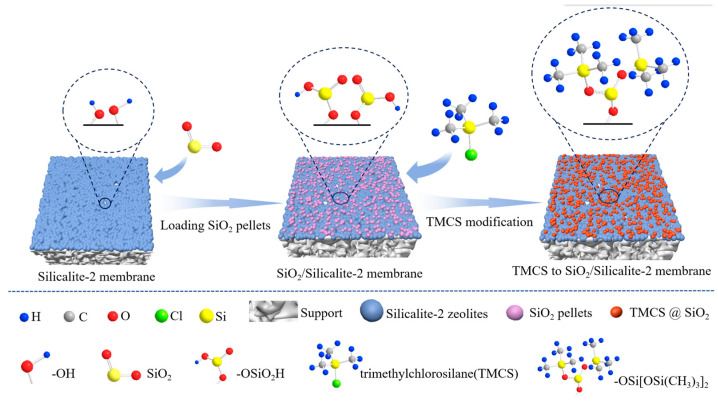
Schematic diagram of Silicalite-2 membrane with TMCS modification.

Figure 12 presents a schematic diagram of Silicalite-2 membranes with TMCS modification. Several SiO_2_ pellets were formed on the surface of Silicalite-2 membranes after treatment with TEOS solution, which increased the number of silica hydroxyl groups on the membrane surface and provided loading sites for TMCS. Thereafter, TMCS reacted with the silica hydroxyl groups and formed -Si-O-Si(CH_3_)_3_ groups on the surface of the membrane, which increased the hydrophobicity of the Silicalite-2 membrane.

## 4. Conclusions

When TBAOH/SiO_2_ and H_2_O/SiO_2_ molar ratios of the precursor synthesis solution were 0.2 and 120, Silicalite-2 membranes were successfully prepared on tubular α-Al_2_O_3_ supports at 150 °C for 72 h by secondary hydrothermal synthesis. The PV performance of membranes could be improved by TMCS modification with medium concentrations. The separation factor and flux of the modified Silicalite-2 membrane were recorded as 22 and 1.75 kg·m^−2^·h^−1^ for 5 wt.% ethanol/H_2_O solution at 60 °C.

## Figures and Tables

**Table 1:** Comparison of Silicalite-2 membranes for the separation performance of 5 wt% ethanol/H_2_O mixtures by pervaporation.

Support	Pervaporation Temperature (°C)	Separation Factor (α_EtOH/H2O_)	Q (kg·m^−2^·h^−1^)	References
α-Al_2_O_3_ hollow fiber	60	17	3.60	[16]
α-Al_2_O_3_ support	60	7	7.61	[17]
α-Al_2_O_3_ support	70	5	1.12	[18]
α-Al_2_O_3_ hollow fiber	60	7	3.30	[19]
α-Al_2_O_3_ supports	70	6	1.28	[20]

**Table 2:** Preparation condition of Silicalite-2 membranes.

No.	Molar Composition of Synthesis Solution			Crystallization Time (h)	Crystallization Temperature (°C)
	TBAOH/SiO_2_	H_2_O/SiO_2_	TMCS Concentration (%)		
C-1	0.15	120	---	72 h	150
C-2	0.20		---		
C-3	0.25		---		
C-4	0.30		---		
C-5	0.20	120	---	72 h	150
C-6		200	---		
C-7		240	---		
C-8	0.20	120	---	48 h	150
C-9			---	72 h	
C-10			---	96 h	
C-11	0.20	120	0	72 h	150
C-12			3.9%		
C-13			5.7%		
C-14			7.5%		

**Table 3:** PV performance of Silicalite-2 membrane and modified Silicalite-2 membranes for 5 wt % EtOH/H_2_O mixture at 60 °C.

Membrane	TMCS Content (wt%)	α_EtOH/H2O_	Q (kg·m^−2^·h^−1^)
C-11	0	3	11.29
C-12	3.9%	19	2.73
C-13	5.7%	22	1.75
C-14	7.5%	17	2.00

## Data Availability

The original contributions presented in the study are included in the article, further inquiries can be directed to the corresponding authors.

## References

[1] Li Y., Tang W., Chen Y., Liu J., Lee C.-F.F. (2019). Potential of acetone-butanol-ethanol (ABE) as a biofuel. Fuel.

[2] Chen Z., He J., Chen H., Geng L., Zhang P. (2021). Comparative study on the combustion and emissions of dual-fuel common rail engines fueled with diesel/methanol, diesel/ethanol, and diesel/n-butanol. Fuel.

[3] Kamarudin M.Z.F., Kamarudin S.K., Masdar M.S., Daud W.R.W. (2013). Review: Direct ethanol fuel cells. Int. J. Hydrogen Energy.

[4] Liu C.-G., Xiao Y., Xia X.-X., Zhao X.-Q., Peng L., Srinophakun P., Bai F.-W. (2019). Cellulosic ethanol production: Progress, challenges and strategies for solutions. Biotechnol. Adv..

[5] Kaseno, Miyazawa I., Kokugan T. (1998). Effect of product removal by a pervaporation on ethanol fermentation. J. Ferment. Bioeng..

[6] Fang L.J., Chen J.H., Yang Q., Lin W.W., Lin Q.J., He Y.S., Zhuo Y.Z. (2022). S-ZIF-8/PEBA/ZIF-8 pervaporation membrane with in situ growing of ZIF-8 active layer on the surface owing outstanding phenol enrichment performance. J. Taiwan Inst. Chem. Eng..

[7] Xu S., Zuo C., Sun X., Ding X., Zhong Z., Xing W., Jin W. (2022). Enriching volatile aromatic compounds of lavender hydrolats by PDMS/ceramic composite membranes. Sep. Purif. Technol..

[8] Luo R., Ding H., Lyu J., Fu T., Bai P., Guo X., Tsapatsis M. (2020). Fabrication of a sandwiched silicalite-1 membrane in a 2D confined space for enhanced alcohol/water separation. Chem. Commun..

[9] Castro-Muñoz R., Boczkaj G. (2021). Pervaporation Zeolite-Based Composite Membranes for Solvent Separations. Molecules.

[10] Wang L., Yang J., Wang J., Raza W., Liu G., Lu J., Zhang Y. (2020). Microwave synthesis of NaA zeolite membranes on coarse macroporous α-Al2O3 tubes for desalination. Microporous Mesoporous Mater..

[11] Wu Z., Peng L., Zhang C., Wang X., Liu H., Wang J., Yan W., Gu X. (2020). Extraction of butanol from ABE solution by MFI zeolite membranes. Sep. Purif. Technol..

[12] Mirfendereski S.M., Lin J.Y.S. (2021). High-performance MFI zeolite hollow fiber membranes synthesized by double-layer seeding with variable temperature secondary growth. J. Membr. Sci..

[13] Lan J., Wu H., Saulat H., Li L., Yang J., Lu J., Zhang Y. (2020). Synthesis of ethanol perm-selective MFI zeolite membranes by binary structure directing agents. J. Membr. Sci..

[14] Peng L., Wu Z., Wang B., Liu H., Zhang C., Gu X. (2022). Fabrication of high-stability W-MFI zeolite membranes for ethanol/water mixture separation. J. Membr. Sci..

[15] Gómez-Álvarez P., Noya E.G., Lomba E. (2022). Structural study of water/alcohol mixtures adsorbed in MFI and MEL porosils. J. Mol. Liq..

[16] Kosinov N., Hensen E.J.M. (2013). Synthesis and separation properties of an α-alumina-supported high-silica MEL membrane. J. Membr. Sci..

[17] Gong L., Zhao M., Chai L.J., Song W.W., Yang J.H. (2021). Preparation of permeable alcohol Silicalite-2 zeolite membranes and their hydrophobic modification. Membr. Sci. Technol..

[18] Wu Q.G., Shao H., Zhong J., Zhang Q., Xu R. (2015). Preparation of Silicalite-2 zeolite membrane and its permeation and vaporization performance. Mod. Chem. Ind..

[19] Kosinov N., Sripathi V.G., Hensen E.J.M. (2014). Improving separation performance of high-silica zeolite membranes by surface modification with triethoxyfluorosilane. Microporous Mesoporous Mater..

[20] Wu Q.-g., Shao H., Zhong J., Zhang Q., Xu R. (2019). Preparation of Sn-silicalite-2 zeolite membranes and their permeation-vaporization properties. Mod. Chem. Ind..

[21] Ren X., Yu H., Guo M., Xu R., Zhong J. (2022). Long alkyl chain-containing organosilica/silicalite-1 composite membranes for alcohol recovery. Microporous Mesoporous Mater..

[22] Kamelian F.S., Mohammadi T., Naeimpoor F., Sillanpää M. (2020). One-Step and Low-Cost Designing of Two-Layered Active-Layer Superhydrophobic Silicalite-1/PDMS Membrane for Simultaneously Achieving Superior Bioethanol Pervaporation and Fouling/Biofouling Resistance. ACS Appl. Mater. Interfaces.

[23] Dib E., Grand J., Gedeon A., Mintova S., Fernandez C. (2021). Control the position of framework defects in zeolites by changing the symmetry of organic structure directing agents. Microporous Mesoporous Mater..

[24] Cheng Y., Pan S. (2013). Preparation and characterization of nanosized silicalite-2 zeolites by steam-assisted dry gel conversion method. Mater. Lett..

[25] Zhang H., Zhang S., Wang H., Li C. (2018). Influence of Initial Water Content on Synthesis of Silicalite-1 Zeolite. China Pet. Process. Petrochem. Technol..

[26] Sousa A.B., Barbosa A.D.S., Rodrigues M.G.F., Laborde H.M. (2012). Preparation and Characterization of Zeolite ZSM-5 in the Template: Effect of Crystallization Time on the Structure and Textural Properties. Mater. Sci. Forum.

[27] Darmawan A., Rasyid S.A., Astuti Y. (2021). Modification of the glass surface with hydrophobic silica thin layers using tetraethylorthosilicate (TEOS) and trimethylchlorosilane (TMCS) precursors. Surf. Interface Anal..

[28] Zemke F., Gonthier J., Scoppola E., Simon U., Bekheet M.F., Wagermaier W., Gurlo A. (2023). Origin of the Springback Effect in Ambient-Pressure-Dried Silica Aerogels: The Effect of Surface Silylation. Gels.

